# Lentinan Alleviated PM2.5 Exposure-Induced Epithelial–Mesenchymal Transition in Pulmonary Epithelial Cells by Inhibiting the GARP/TGF-β/Smad Pathway

**DOI:** 10.3390/toxics13030166

**Published:** 2025-02-26

**Authors:** Zhi Wang, Shiqing Xu, Bohao Bian, Zhida Hu, Feiyang Wu, Siqi Zhao, Xiaohui Wang, Li Wang, Teng Ma

**Affiliations:** 1 School of Public Health, Baotou Medical College, Inner Mongolia University of Science & Technology, Baotou 014040, China; 2022200104@stu.btmc.edu.cn (Z.W.); 2022200097@stu.btmc.edu.cn (S.X.); 17302256519@163.com (F.W.); sqtjzy@163.com (S.Z.); 102018909@btmc.edu.cn (X.W.); 2 Hulunbuir Center for Disease Control and Prevention, Hulun Buir 021000, China; 13604749342@163.com; 3 Cangzhou People’s Hospital, Department of Hospital Infection Management, Cangzhou 061000, China; 2021400116@stu.btmc.edu.cn

**Keywords:** COPD, PM2.5, lentinan, GARP, EMT

## Abstract

PM2.5 (fine particulate matter) is an air pollutant widely present in urban and industrial areas, which has emerged as a significant threat to human health. Specifically, long-term exposure to PM2.5 could lead to various lung diseases, including pulmonary fibrosis and Chronic Obstructive Pulmonary Disease (COPD). The Glycoprotein A Repetitions Predominant (GARP) protein, a key receptor and regulator for TGF-β1, has recently emerged as a vital cytokine in PM2.5-induced pulmonary pathological changes. As a membrane glycoprotein, GARP binds to TGF-β, keeping it in an active state. Herein, PM2.5 treatment upregulated GARP and promoted Epithelial–Mesenchymal Transition (EMT) via TGF-β/SMAD signaling pathway activation. Conversely, lentinan (a shiitake mushroom-derived polysaccharide) effectively reversed the PM2.5-induced GARP upregulation, alleviating EMT. This study elucidates the role of GARP in PM2.5-induced EMT through the TGF-β/SMAD pathway in pulmonary epithelial cells and discusses the therapeutic potential of lentinan.

## 1. Introduction

Lung diseases account for a substantial proportion of morbidities and mortalities worldwide, posing significant health risks [1]. Air pollution—especially by fine particulate matter (PM2.5)—is among the many factors that contribute to the development of respiratory illnesses [2]. Owing to the fact that they are small enough to penetrate deep into the lungs and enter the bloodstream, PM2.5 particles have been linked to various respiratory conditions, ranging from chronic bronchitis to more severe complications such as emphysema and lung fibrosis [3,4,5]. According to the research, long-term exposure to PM2.5 could exacerbate inflammation in the airways [6], triggering symptoms such as coughing [7], wheezing [8], and shortness of breath [9]. Moreover, prolonged PM2.5 exposure could increase the risk of developing more severe lung conditions, with its impact also extending to the cardiovascular system, further complicating overall health [10]. Therefore, given the ubiquity of PM2.5 in urban and industrial areas, addressing air pollution could be crucial to reducing the burden of lung diseases and improving public health.

The specific mechanism of PM2.5 exposure in lung diseases involves the exacerbation of airway inflammation and remodeling, which activates Epithelial–Mesenchymal Transition (EMT) processes in lung epithelial cells [11]. The cellular alterations that follow promote airway obstruction and pulmonary fibrosis, which are hallmarks of Chronic Obstructive Pulmonary Disease (COPD) [12]. According to research, EMT contributes to the loss of epithelial integrity, elevating the risk of respiratory infections and causing a further decline in lung function [13]. Therefore, understanding the mechanisms through which PM2.5 mediates EMT could provide crucial insights into the treatment of lung diseases. Regarding tumorigenesis, PM2.5 could also induce chronic inflammation and Oxidative Stress (OS), which are crucial factors that promote EMT in various cancer types [14]. Furthermore, PM2.5 exposure could trigger signaling pathways such as TGF-β and NF-κB [15], a transition that enhances cancer cell migratory and invasive capabilities, contributing to metastasis and a poor prognosis [16,17].

Lentinan, a polysaccharide derived from the cell wall of shiitake mushrooms, could strengthen the host immune system, helping in preventing various illnesses. Lentinan has been shown to exert anti-inflammatory effects via immune response modulation [18]. Specifically, it could inhibit the secretion of pro-inflammatory cytokines such as TNF-α, IL-6, and IL-1β, thus reducing inflammation. Lentinan achieves this effect through the regulation of key signaling pathways, including the NF-κB and MAPK pathways, which are crucially involved in inflammatory responses [19]. Research has reported lentinan’s potential in alleviating chronic inflammatory illnesses, such as Rheumatoid Arthritis (RA) and Inflammatory Bowel Disease (IBD), highlighting its potential utility as an adjunctive therapy to conventional anti-inflammatory treatments [20]. The anti-inflammatory properties of lentinan could also help reduce airway hyper-responsiveness and tissue damage in asthma and COPD patients. Additionally, lentinan could play an anti-tumor role by alleviating inflammatory response and reducing the production of EMT [21]. Nonetheless, research on the role of lentinan in alleviating air pollutant-induced EMT is relatively scarce.

The Glycoprotein A Repetitions Predominant (GARP) protein is a transmembrane protein that regulates TGF-β on the cell surface by binding to it, thus activating the TGF-β signaling pathway [22]. In cancer, GARP enhances TGF-β activation, thus promoting EMT, which, in turn, leads to increased tumor cell migration and invasion, ultimately promoting tumor progression and metastasis [23]. Moreover, GARP upregulation has been associated with immune evasion in various malignancies, further supporting tumor growth [24,25]. In respiratory diseases, particularly COPD and asthma, GARP upregulation might contribute to airway remodeling and fibrosis via TGF-β-mediated EMT, resulting in airway obstruction and reduced lung function [26]. Furthermore, GARP could influence inflammatory responses within the airways, exacerbating symptoms of pertinent conditions [27]. However, the role of GARP in PM2.5-induced lung diseases remains unclear. Although the role of GARP in immune modulation and cancer has been extensively studied, its involvement in particulate matter-induced EMT, particularly in respiratory diseases, remains largely unexplored. This study is the first to identify GARP as a key regulator of PM2.5-induced EMT and to investigate the therapeutic potential of lentinan, in mitigating this process. The results offer new perspectives on PM2.5’s pathological effects on lungs, suggesting GARP’s promise in treating airway illnesses.

## 2. Materials and Methods

### 2.1. Cell Culture and PM2.5 Sampling and Preparation

Beas-2B human bronchial epithelial cells were obtained from Pricella Life Science and Technology Co., Ltd. (Wuhan, China). The cell culture was maintained in Dulbecco’s Modified Eagle Medium (DMEM) (Gibco, Gaithersburg, MD, USA). To create the complete medium, we supplemented the DMEM with 10% Fetal Bovine Serum (FBS) (Gibco, Gaithersburg, MD, USA) and a 1% antibiotic solution [containing 100 IU/mL penicillin and 100 μg/mL streptomycin (Beyotime, Shanghai, China)]. The cells were grown in a humidified incubator (Thermo Scientific, Waltham, MA, USA), maintaining a temperature of 37 °C and a 5% CO_2_ atmosphere. Upon reaching 80–90% confluency, the Beas-2B cells were trypsinized to detach them, then replated uniformly in well plates or culture dishes. Subsequently, these cells were exposed to varying concentrations of PM2.5 culture solution for a 24 h period before being harvested for further experimentation.

Herein, PM2.5 was sampled 1.5 m above the ground on the roof of Baotou Medical College’s laboratory building using an air sampler KB-6210 with a flow rate of 100 L/min. The sampling lasted 21 h (from 9:00 to 6:00 the next day), and the sampling temperature, air pressure, and site conditions were recorded. The collected samples were folded in half twice using tweezers, placed in tinfoil, and balanced in a dryer for 24 h. We previously conducted a chemical analysis of the composition of PM2.5. Research showed that rare earth elements exhibit a strong positive correlation with inorganic elements, indicating a consistent source or shared anthropogenic influences, primarily from coal and oil combustion as well as industrial pollution. The high enrichment factors of Se, Cd, Ag, Pb, and As in PM2.5 suggest that their origins are largely attributed to anthropogenic pollution, with minimal influence from crustal sources [28]. After removing the filter membrane, the samples were weighed with the same electronic balance as before sampling, and stored in a refrigerator at 20 °C, awaiting further tests. Subsequently, the particles were dissolved in DMEM at a concentration of 1000 μg/mL and stored at 4 °C in the laboratory, awaiting further study. Finally, the cells were treated with different concentrations of PM2.5 (0, 6.25, 12.5, and 25 μg/mL) for 24 h.

### 2.2. Cell Proliferation Assay

Cell viability was evaluated utilizing the Cell Counting Kit-8 (CCK-8) assay (Biosharp, Hefei, China). In summary, BEAS-2B cells were plated at a density of 5000 cells per well in 96-well plates and exposed to different concentrations of PM2.5 for 24 h. For the experimental group, the culture medium was continuously refreshed with a new solution containing lentinan. After the incubation period, the old medium was discarded and substituted with an equal volume of fresh medium enriched with 10% CCK-8 reagent. The cells were then further incubated for 2 h at 37 °C. Ultimately, the absorbance was recorded at 450 nm using a microplate reader (Multiskan MK3, Thermo Fisher Scientific, Waltham, MA, USA).

### 2.3. Wound Healing Assay (WHA)

First, Beas-2B cells from each group were inoculated into 6-well plates. At 70–80% growth, a straight line was evenly drawn in the pores with a 200 μL gun tip. After cleaning the cell fragments with PBS, a serum-free medium was added. The images of cells were observed at 0 and 24 h and photographed using a microscope. The scratch area was quantified using ImageJ version 1.50 software.

### 2.4. Transwell Assay (TWA)

Cellular invasion was evaluated using a Transwell chamber setup. Initially, Matrigel was mixed with DMEM at a dilution of 1:8. This blend was then placed into cold Transwell chambers (8 μm pore size, Corning, New York, NY, USA) and left to incubate at 37 °C for 2 h to facilitate gel solidification. Subsequently, a cell suspension at a concentration of 1 × 10^5^/mL was prepared, and 100 µL of this suspension was introduced into the upper chamber containing serum-free medium. Meanwhile, the lower chamber was filled with 700 µL of DMEM enriched with 10% FBS. After a 48 h incubation period, cells that migrated to the lower side of the membrane were counted using an inverted microscope and stained with crystal violet. To fix the cells, a 4% Paraformaldehyde (PFA) solution was applied for 15 min.

### 2.5. Real-Time Polymerase Chain Reaction (RT-PCR)

Herein, RT-PCR was performed using the Applied Biosystems GeneAmp 9700 PCR system (Applied Biosystems), with human GAPDH employed as an internal control. All real-time data were analyzed using the comparative Ct method and normalized to GAPDH. The primers for the amplification of GARP cDNA were as follows: forward (5′–3′): GCATAGCAACGTGCTGATGGAC; reverse (5′–3′): GATGCTGTTGCAGCTCAGGTCT. The primers for the amplification of TGF-β1 cDNA were as follows: forward (5′–3′): TACCTGAACCCGTGTTGCTCTC; reverse (5′–3′): GTTGCTGAGGTATCGCCAGGAA. The primers used to amplify GAPDH were as follows: forward (5′–3′): ACAACTTTGGTATCGTGGAAGG; reverse (5′–3′): GCCATCACGCCACAGTTTC.

### 2.6. Western Blot (WB) Assay

Cells were collected, and protein levels were quantified via the BCA assay kit (Beyotime, Beijing, China). Following this, proteins underwent separation using SDS-PAGE, which were subsequently transferred onto PVDF membranes (Millipore, Burlington, NJ, USA). The membranes were then blocked with 5% non-fat milk for a duration of 2 h before being incubated overnight at 4 °C with primary antibodies targeting Vimentin (1:1000; Affinity, Cincinnati, OH, USA), E-Cadherin (1:1000; Affinity, Cincinnati, OH, USA), N-Cadherin (1:1000; Affinity, Cincinnati, OH, USA), GARP (1:1000; Proteintech, Wuhan, China), TGF-β1 (1:800; Abcam, Cambridge, UK), p-SMAD2/3 (1:1000; Abcam, Cambridge, UK), and β-Actin (1:10,000; Affinity, Cincinnati, OH, USA). After this incubation, the membranes were treated with HRP-conjugated secondary antibodies (1:5000; Affinity, Cincinnati, OH, USA) at room temperature for an hour. Lastly, the membranes were visualized using ECL reagent (APPLYGEN, Beijing, China).

### 2.7. RNA Interference

GARP inhibitor (si-GARP) and si-GARP Negative Control (si-NC) were sourced from IGEBio (Guangzhou, China). Liposome 2000 reagent (Invitrogen) was used to complete the transfection. Briefly, the cells were first inoculated onto a six-well plate. Afterward, a MEM solution infused with either si-GARP or si-NC was combined with another MEM solution containing Liposome 2000 reagent. This concoction was then employed to transfect the cells over a 6 h period. Once the transfection was complete, the cells were subjected to PM2.5 exposure for 24 h, after which they were harvested and stored, ready for subsequent experimental procedures.

### 2.8. Statistical Analysis

Each experiment was conducted three separate times. We used GraphPad Prism 9.0 for all statistical analyses. When comparing the two groups, we ran a Student’s *t*-test. For comparisons involving more than two groups, we used a one-way ANOVA followed by Tukey’s post hoc test. We considered results with * *p* < 0.05 to be statistically significant, and those with ** *p* < 0.01 to be highly significant.PM2.5

## 3. Results

### 3.1. Lentinan Reversed the PM2.5-Induced Decrease in Cell Activity

**Figure 1:**
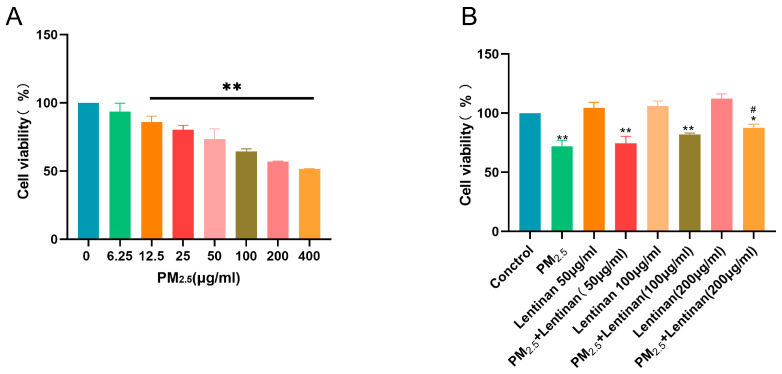
Lentinan reversed the PM2.5-induced decreased cell activity: (**A**) CCK-8-detected effect of different concentrations of PM2.5 on Beas-2B cell viability after 24 h of treatment; and (**B**) CCK-8-assessed protective effect of lentinan onPM2.5-induced cell damage. Compared to the control group, *: *p* < 0.05, **: *p* < 0.01. Compared to the PM2.5 group, #: *p* < 0.05. n = 3.

Following exposure to varying levels of PM2.5 and lentinan, cell viability was evaluated using the CCK-8 assay. The findings revealed that, when compared to the control group, cells treated with a PM2.5 concentration of 12.5 μg/mL showed a notable decline in proliferation activity (*p* < 0.01) (Figure 1A). For subsequent experiments, PM2.5 doses of 6.25, 12.5, and 25 μg/mL were chosen as the focus groups. In contrast, while treatment with 50/100 μg/mL lentinan failed to counteract the diminished cell viability caused by PM2.5 exposure, a significant improvement was observed at a lentinan concentration of 200 μg/mL (*p* < 0.05) (Figure 1B). As a result, 200 μg/mL lentinan was selected for further experimental treatments.

### 3.2. PM2.5 Promoted Invasive, Migratory, and EMT Induction Abilities in Beas-2B Cells

**Figure 2:**
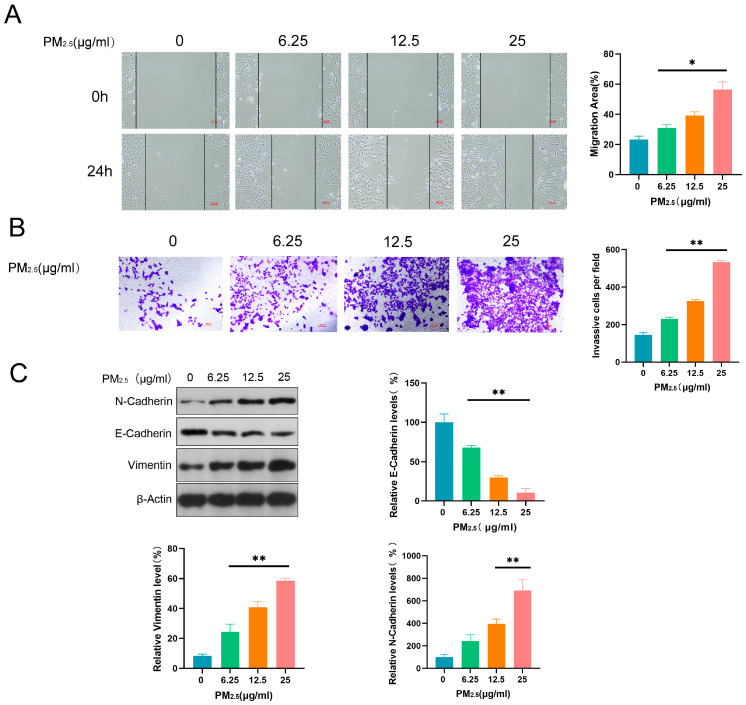
PM2.5 enhanced the invasive, migratory, and EMT induction abilities of Beas-2B cells. (**A**) WHA-detected migratory ability of Beas-2B cells; (**B**) TWA-detected invasive ability of Beas-2B cells; and (**C**) WB-detected expression of EMT-related proteins N-Cadherin, E-Cadherin, and Vimentin. *: *p* < 0.05, **: *p* < 0.01.

After treatment with different concentrations of PM2.5 (0, 6.25, 12.5, and 25 μg/mL) for 24 h, Beas-2B cells were assessed for their migratory, invasive, and EMT induction abilities using WHA, TWA, and WB. Compared to the control group, Beas-2B cells exposed to PM2.5 exhibited higher migratory and invasive abilities (Figure 2A,B). Furthermore, PM2.5 significantly upregulated N-Cadherin and Vimentin, and downregulated E-Cadherin, in a dose-dependent manner, suggesting that it induced the EMT process in Beas-2B cells (Figure 2C).

### 3.3. PM2.5 Activated the GARP/TGF-β/Smad Pathway

**Figure 3:**
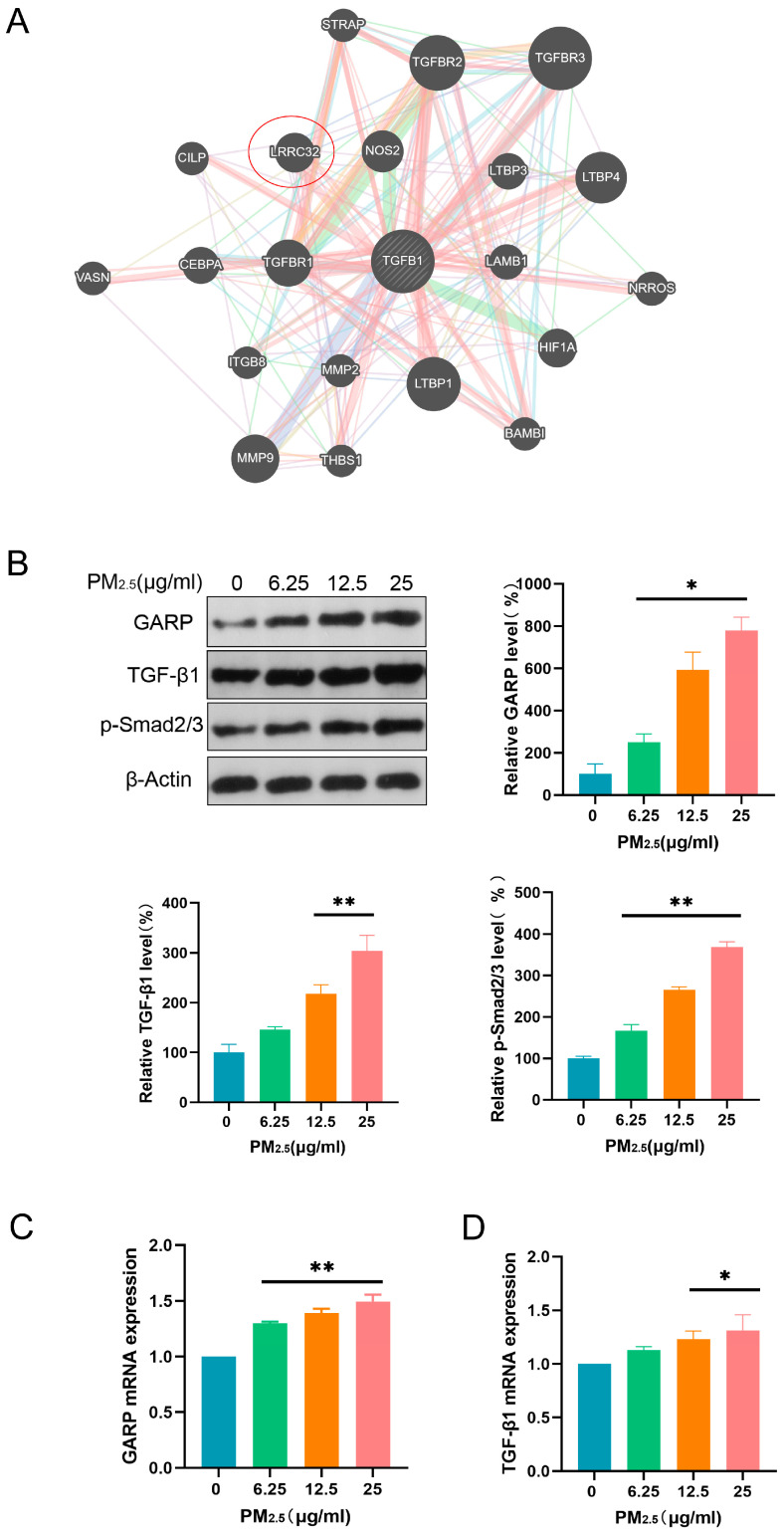
PM2.5 activated the GARP/TGF-β/Smad pathway: (**A**) Protein binding was predicted using Genemania databases; (**B**) WB analysis of GARP/TGF-β/Smad pathway-associated proteins; (**C**) PCR-detected relative GARP mRNA expression; and (**D**) PCR-detected relative TGF-β1 mRNA expression. *: *p* < 0.05, **: *p* < 0.01.

Herein, network-based predictions were performed using Genemania databases to investigate the potential interaction between GARP and TGF-β. We found that GARP (LRRC32) could interact with TGF-β, implying the former’s direct or indirect involvement in the regulation of the TGF-β/SMAD pathway under PM2.5 exposure (Figure 3A). To establish whether PM2.5 impacted the GARP/TGF-β/Smad pathway, proteins related to this pathway were detected through WB after exposure to PM2.5 for 24 h. According to the results, the GARP, TGF-β1, and p-Smad2/3 proteins in the cells were significantly upregulated after exposure to PM2.5 (Figure 3B). Subsequently, the mRNA contents of GARP and TGF-β1 were detected using PCR, revealing that the mRNA expression levels of GARP and TGF-β1 increased with the PM2.5 exposure dose (Figure 3C,D). These findings suggest that PM2.5 can activate the GARP/TGF-β/Smad pathway.

### 3.4. GARP Knockdown Reversed the Effects of PM2.5 on Beas-2B Cell Migratory, Invasive, and EMT Induction Abilities

**Figure 4:**
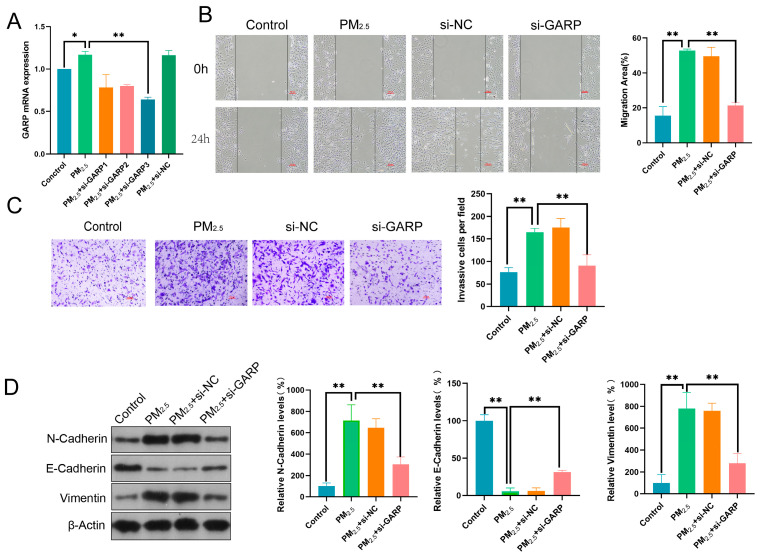
GARP knockdown reversed the effects of PM2.5 on Beas-2B cell migratory, invasive, and EMT induction abilities: (**A**) PCR-detected GARP expression; (**B**) WHA-detected migratory ability of Beas-2B cells; (**C**) TWA-detected invasive ability of Beas-2B cells; and (**D**) WB-detected expression of EMT-related proteins N-Cadherin, E-Cadherin, and Vimentin. *: *p* < 0.05, **: *p* < 0.01.

To establish whether the GARP/TGF-β/Smad axis correlated with the effect of PM2.5 on Beas-2B cells, we successfully constructed a GARP knockdown cell model (Figure 4A). According to the results, GARP downregulation inhibited the migratory and invasive abilities of PM2.5-exposed cells (Figure 4B,C). Furthermore, N-Cadherin and Vimentin were significantly downregulated, while E-Cadherin was upregulated, suggesting that inhibiting the GARP/TGF-β/Smad pathway could reverse the PM2.5-induced EMT process in Beas-2B cells (Figure 4D).

### 3.5. Lentinan Mitigated the Impact of PM2.5 on Beas-2B Cells

**Figure 5:**
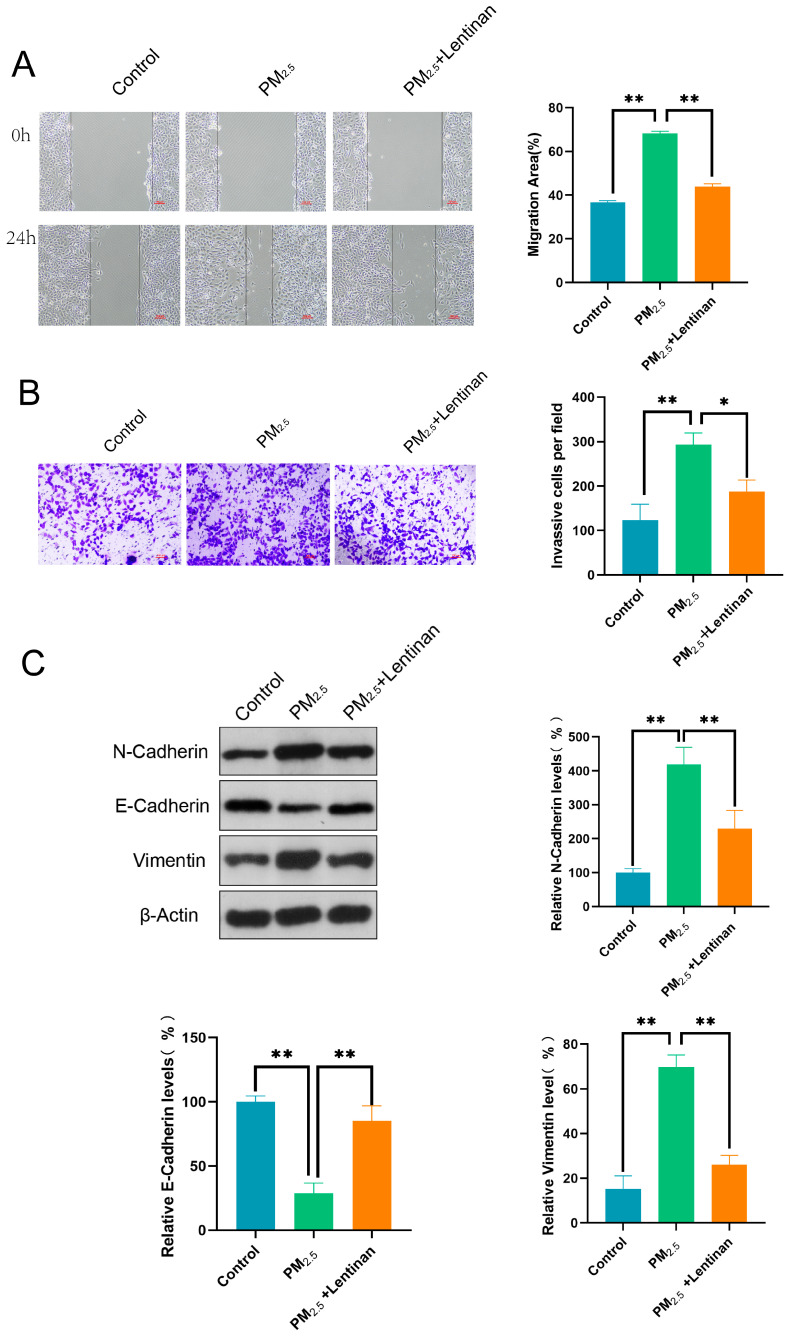
Lentinan inhibited the PM2.5-induced migratory, invasive, and EMT induction abilities of Beas-2B cells: (**A**) WHA-detected migratory ability of Beas-2B cells; (**B**) TWA-detected invasive ability of Beas-2B cells; and (**C**) WB-detected expression of EMT-related proteins N-Cadherin, E-Cadherin, and Vimentin. *: *p* < 0.05, **: *p* < 0.01.

Herein, Beas-2B cells were exposed to PM2.5 for 24 h and then treated with lentinan for an additional 24 h to establish whether the latter treatment could mitigate the effect of PM2.5 on the cells. Subsequently, the migratory, invasive, and EMT induction abilities of the cells were detected using WHA, TWA, and WB. According to the results, lentinan effectively suppressed the migratory and invasive properties of cells exposed to PM2.5 (Figure 5A,B). Additionally, in comparison to the PM2.5 group, treatment with lentinan led to a marked decrease in the expression of N-Cadherin and Vimentin, while simultaneously increasing the expression of E-Cadherin. This suggests that lentinan has the potential to reverse the EMT process induced by PM2.5 in Beas-2B cells (Figure 5C).

### 3.6. Lentinan Inhibited the PM2.5-Induced GARP/TGF-β/Smad Pathway Activation

**Figure 6:**
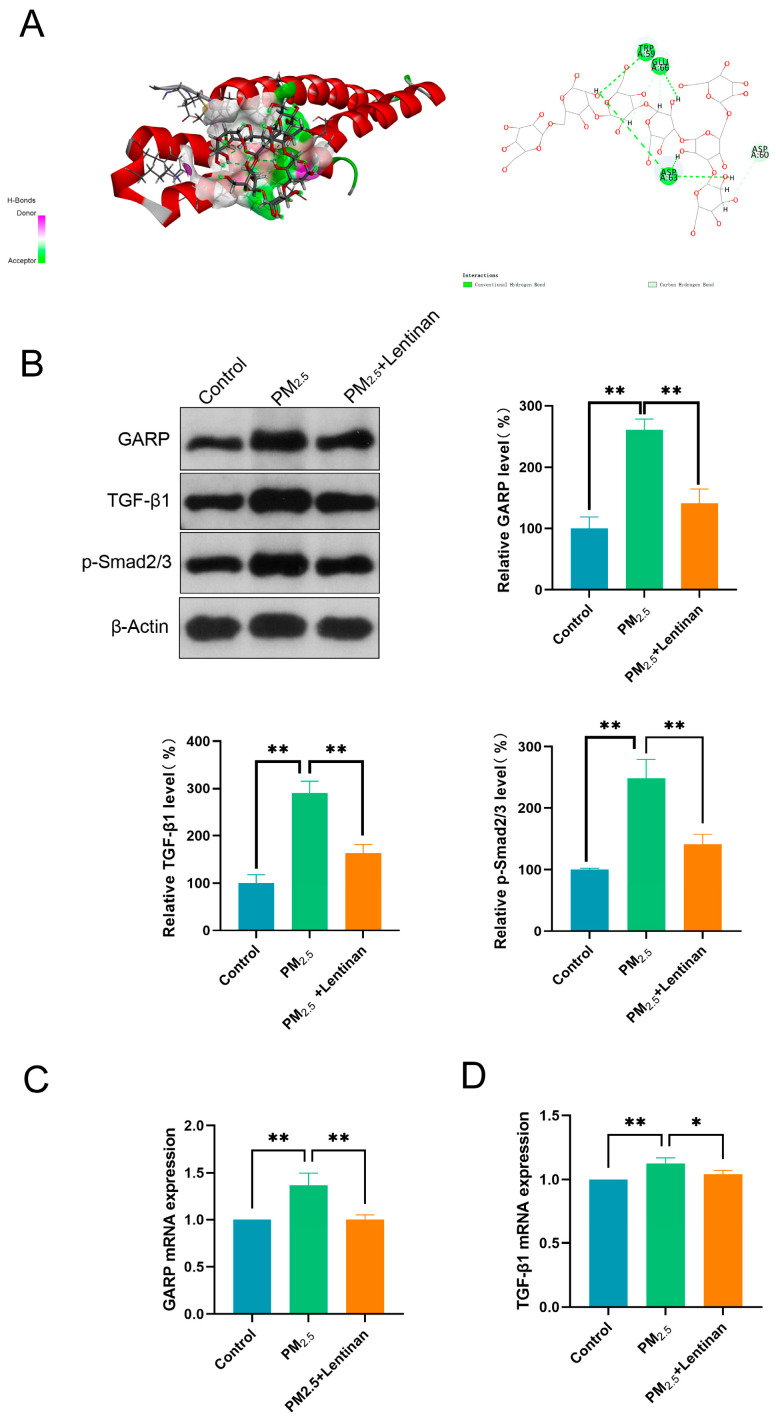
Lentinan inhibited the PM2.5-induced GARP/TGF-β/Smad pathway activation: (**A**) Docking of Lentinan with GARP; (**B**) WB analysis of GARP/TGF-β/Smad pathway-associated proteins; (**C**) PCR-detected relative GARP mRNA expression; and (**D**) PCR-detected relative TGF-β1 mRNA expression. *: *p* < 0.05, **: *p* < 0.01.

**Figure 7:**
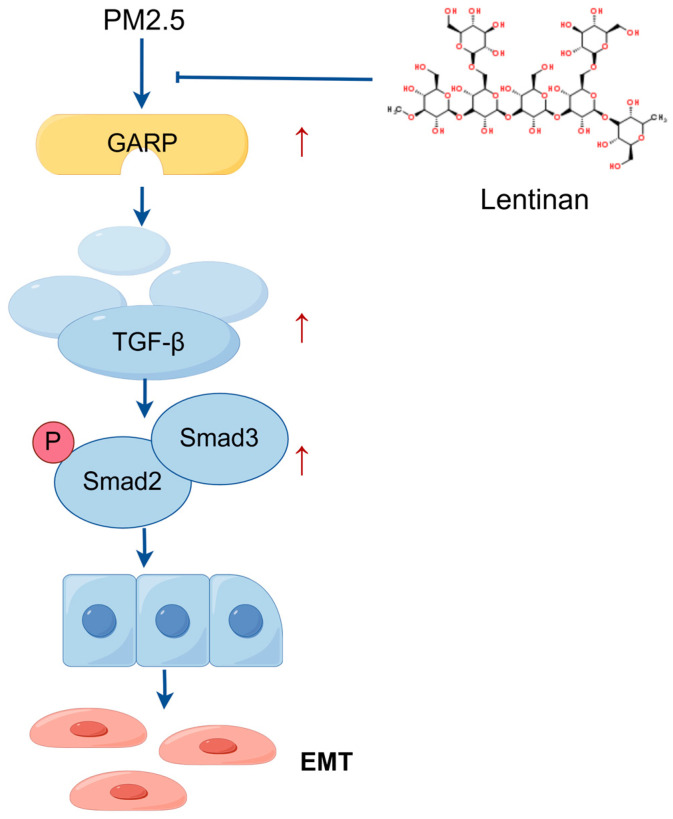
Possible mechanism of lentinan in alleviating the PM2.5-induced EMT process in Beas-2B cells. PM2.5 can upregulate GARP, activating TGF-β, thus promoting the phosphorylation of Smad2 and Smad3 and activating the GARP/TGF-β/Smad pathway, which, in turn, induces EMT in Beas-2B cells. Lentinan inhibited GARP production and GARP/TGF-β/Smad pathway activation, thus improving the PM2.5-induced EMT (Figdraw).

To establish whether lentinan treatment could impact the GARP/TGF-β/Smad pathway in PM2.5-exposed cells, potential EMT targets and major compounds were subjected to molecular docking analysis using Discovery Studio CDOCKER. The main extract of lentinan was selected for docking with key GARP targets, revealing that the ligands and receptors exhibited binding properties (Figure 6A). Proteins associated with the GARP/TGF-β/Smad pathway were detected through WB analysis 24 h after lentinan treatment. According to the results, the expression of the GARP, TGF-β1, and p-Smad2/3 proteins was significantly lower post-treatment compared to the PM2.5 exposure group (Figure 6B). Subsequently, GARP and TGF-β1 mRNA levels were detected using PCR, revealing that the mRNA expression levels of GARP and TGF-β1 were lower compared to those in the PM2.5-exposed group (Figure 6C,D). These findings suggest that lentinan inhibited the PM2.5-induced GARP/TGF-β/Smad pathway activation. Thus, it is demonstrated that Lentinan alleviated PM2.5 exposure-induced epithelial–mesenchymal transition in pulmonary epithelial cells by inhibiting the GARP/TGF-β/Smad pathway (Figure 7).

## 4. Discussion

Numerous studies have highlighted the crucial involvement of PM2.5 in triggering EMT, which promotes tissue remodeling, fibrosis, and metastasis [29,30]. Due to their small size and chemical composition, PM2.5 particles can penetrate deeply into the respiratory system, inducing OS [31], inflammation [32], and alterations in cellular signaling [33]. One of the key mechanisms through which PM2.5 induces EMT is via TGF-β signaling pathway activation. This pathway, often dysregulated by environmental pollutants such as PM2.5, promotes the expression of mesenchymal markers (e.g., N-Cadherin, vimentin) and suppresses epithelial markers (e.g., E-Cadherin), thus triggering EMT [34]. Therefore, PM2.5 exposure could drive EMT in various models of respiratory diseases such as COPD and asthma, as well as in cancer [35], contributing to airway fibrosis [36], lung damage [37], and tumor metastasis [38]. In other words, PM2.5 exposure could accelerate the progression of both respiratory diseases and cancer through EMT.

Our study reveals a previously unreported role of GARP in promoting EMT in relation to PM2.5 exposure. Notably, GARP has been largely studied in the context of immune modulation and TGF-β activation. Consequently, our findings on its role in EMT, particularly in respiratory cells and under conditions such as PM2.5 exposure, offer novel insights into its potential as a therapeutic target. Specifically, we found that GARP enhances TGF-β activation, which, in turn, induces the expression of EMT markers and facilitates the transition from an epithelial to a mesenchymal phenotype in lung ECs. This finding positions GARP as a novel molecular mediator of PM2.5-induced EMT, suggesting that targeting GARP could be an avenue to mitigating the deleterious effects of PM2.5 exposure in respiratory diseases and other related conditions.

To further elucidate the mechanisms by which GARP regulates PM2.5-induced EMT, it is essential to explore both Smad-dependent and Smad-independent pathways. GARP, as a key regulator of TGF-β activation, may influence the phosphorylation of Smad2 and Smad3, which are critical downstream effectors of the TGF-β signaling pathway. The activation of Smad2/3 can lead to the upregulation of transcription factors such as Snail, Twist, and ZEB1, which are known to promote EMT by repressing epithelial markers and enhancing mesenchymal markers [39]. Future studies should investigate whether GARP directly interacts with Smad proteins or if it modulates other signaling molecules that crosstalk with the TGF-β pathway, such as MAPK/ERK or PI3K/Akt. Additionally, the role of GARP in regulating non-canonical TGF-β signaling pathways, such as those involving RhoA or JNK, should be explored to provide a more comprehensive understanding of its regulatory mechanisms.

Moreover, the interaction between GARP and other cellular components, such as integrins or extracellular matrix proteins, could also play a role in PM2.5-induced EMT [40,41]. GARP may facilitate the activation of latent TGF-β by binding to integrins, which are known to be involved in the mechanical transduction of signals from the extracellular environment. This interaction could be particularly relevant in the context of PM2.5 exposure, as the physical properties of particulate matter may alter the mechanical forces within the lung tissue, thereby influencing EMT. Further research should aim to identify the specific integrins or other cell surface receptors that interact with GARP and determine how these interactions contribute to the activation of TGF-β and subsequent EMT.

Although GARP has been identified as a key player in PM2.5-induced EMT, it is noteworthy that other molecules and signaling pathways could also contribute to this process. Potential candidates include TGF-β receptors, Smad proteins, and TFs (such as Snail, Twist, and ZEB1), which are known to regulate EMT in various disease models. Furthermore, PM2.5 could activate the MAPK/ERK and PI3K/Akt pathways [42], which are involved in inflammation and cell survival, thus contributing to EMT. Therefore, targeting these molecules could provide alternative therapeutic avenues for alleviating the harmful effects of PM2.5 exposure.

Our findings also revealed that lentinan could alleviate PM2.5-induced EMT, a phenomenon attributable to its ability to inhibit the GARP signaling pathway and reduce OS. Lentinan could exert its effects through various mechanisms including inhibiting inflammation pathway activation [43], reducing Reactive Oxygen Species (ROS) production [44], and suppressing the expression of key Transcription Factors (TF) that regulate EMT (such as Snail and Twist). Additionally, lentinan may protect cells by chelating toxic metals present in PM2.5, thereby reducing their bioavailability and cellular toxicity. Polysaccharides have been shown to bind metal ions, which could prevent their interaction with cellular components. Future studies should explore whether lentinan’s protective effects are partially mediated through this mechanism [45]. Nonetheless, hitherto, lentinan’s molecular mechanism in protecting against PM2.5 exposure was unreported. Furthermore, the bioavailability of lentinan in vivo is an important consideration. Polysaccharides like lentinan may be degraded by gut microbiota into smaller oligosaccharides, which are more easily absorbed through the intestinal mucosa. This raises the possibility that the protective effects of lentinan observed in vitro may be mediated by its metabolites rather than the intact polysaccharide [46]. For example, β-1,3-oligosaccharides derived from polysaccharides have been shown to activate monocytes and release TNF-α, which may contribute to their anti-inflammatory and protective effects [47]. Future studies should explore the metabolic pathways of lentinan in vivo, its bioavailability, and compare the efficacy of polysaccharides and oligosaccharides in mitigating PM2.5-induced EMT.

In light of its findings, this study offers several valuable contributions to the medical field. First, it identifies GARP as a novel regulator of PM2.5-induced EMT, highlighting its involvement in TGF-β activation and its potential as a therapeutic target. Furthermore, it provides novel insights into the mechanisms through which lentinan can mitigate PM2.5-induced EMT, presenting a potentially natural therapeutic strategy for counteracting the adverse effects of air pollution on lung health. Overall, our study lays the groundwork for additional research into GARP-targeting therapies and natural compounds for respiratory disease treatment.

Despite its valuable insights regarding GARP’s role in PM2.5-induced EMT, this study had certain limitations. For instance, the in vivo relevance of our findings in animal models of PM2.5 exposure remains to be fully established. This study was primarily based on in vitro cell models, and the translation of its findings to in vivo models could be challenging. Additionally, although our in vitro data suggest that GARP is crucially involved in EMT mediation, we did not explore the precise molecular mechanisms linking GARP activation to the TGF-β signaling pathway, necessitating additional research for a deeper mechanistic understanding of how GARP influences the Smad-dependent and Smad-independent pathways in response to PM2.5 exposure to validate its therapeutic potential. Moreover, we did not explore the long-term effects of lentinan treatment in chronic PM2.5 exposure models. This study did not explore the broader impact of GARP inhibition or lentinan treatment on the immune system, as well as the potential side effects. Finally, we have only provided preliminary insights into the mechanisms of EMT, necessitating further detailed molecular profiling and validation in animal models to confirm our findings and refine the potential therapeutic approaches.

Future studies should focus on elucidating the precise molecular mechanisms linking GARP activation to both Smad-dependent and Smad-independent pathways in response to PM2.5 exposure. Specifically, CRISPR/Cas9-mediated GARP knockout models and RNA-seq analysis could provide deeper insights into the regulatory networks involved. Additionally, in vivo studies using animal models of PM2.5 exposure are needed to validate the therapeutic potential of lentinan and GARP-targeting strategies. Furthermore, the synergistic effects of PM2.5 with other environmental pollutants, such as microplastics and nanoparticles, should be explored to better understand the broader implications of air pollution on lung health.

Beyond the molecular mechanisms explored herein, it is also essential to consider the broader implications of PM2.5-induced EMT regarding environmental health and disease prevention. Presently, PM2.5 exposure is a global concern due to its pervasiveness in urban areas, as well as its association with a range of respiratory and cardiovascular diseases. Therefore, understanding the molecular players involved in EMT, such as GARP, offers critical insights into the pathophysiology of air pollution-related diseases. Moreover, targeting GARP and other key regulators of EMT offers a promising avenue for developing novel therapeutic strategies, especially in the face of increasing pollution levels worldwide. Furthermore, natural compounds like lentinan, which exert protective effects against PM2.5-induced EMT, could serve as adjuncts to contemporary therapeutic options, offering a more holistic approach to combating the deleterious effects of air pollution. There is also a need to explore the potential synergistic effects of combining GARP inhibition agents with other pharmacological or lifestyle interventions in mitigating the adverse impacts of PM2.5 exposure on public health.

## 5. Conclusions

This article highlights the critical role of PM2.5 in EMT induction and the novel function of GARP in this process, as well as the potential therapeutic benefits of lentinan. In conclusion, this study identifies GARP as a novel regulator of PM2.5-induced EMT and highlights the therapeutic potential of lentinan in mitigating this process. Our findings provide new insights into the molecular mechanisms underlying PM2.5-induced lung damage, and they lay the groundwork for future research into GARP-targeting therapies and natural compounds for the treatment of respiratory diseases. Moreover, we emphasize the need for further investigation into unresolved questions, the identification of additional therapeutic targets, and the in vivo validation of this study’s findings. Overall, this study presents a step forward in understanding the molecular mechanisms underlying PM2.5-induced lung damage and lays the groundwork for future research into EMT-targeting therapeutic strategies for respiratory illnesses.

## Data Availability

The data that support the findings of this study are available from the corresponding author upon reasonable request.
